# Effect of Hematoporphyrin Monomethyl Ether-Sonodynamic Therapy (HMME-SDT) on Hypertrophic Scarring

**DOI:** 10.1371/journal.pone.0086003

**Published:** 2014-01-21

**Authors:** Hanjun Zhang, Xing Liu, Youbin Liu, Yin Wu, Hongxi Li, Chengbin Zhao, Huazhe Li, Qinggang Meng, Wei Li

**Affiliations:** 1 Department of Orthopaedic Surgery, Harbin Medical University, Harbin, China; 2 Department of Orthopaedic Surgery, The First Hospital of Harbin City, Harbin, China; University of Arkansas for Medical Sciences; College of Pharmacy, United States of America

## Abstract

**Objective:**

The aim of the present study was to explore the potential for hematoporphyrin monomethyl ether-Sonodynamic Therapy (HMME-SDT) treatment of hypertrophic scars within rabbit ears.

**Methods:**

60 white rabbits were randomly divided into five groups: 1) untreated controls, 2) lesioned, 3) lesioned + HMME, 4) lesioned + US (Ultrasound), and 5) lesioned +HMME-SDT. After induction of a lesion upon the ears of the rabbits, hypertrophic scars were assessed at 14, 28, 42 and 56 days post-lesion +/− treatment. Assessments consisted of visual inspection in the change of the skin, scar formation pathological morphology by hematoxylin and eosin (HE) staining technique with optical microscopy, calculation of a hypertrophic index, fibroblastic density measures, and observation of collagen changes in the scar tissue by Van Gieson's (VG)Stain along with calculation of collagen area density.

**Results:**

With continued HMME-SDT treatment there was a gradual improvement in all parameters over the duration of the experiment. The lesion-induced scars of rabbits receiving HMME-SDT treatment were soft, the size was reduced, hyperplasia was flat and the color pale. The fibroblasts and collagens were reduced and the collagens were light red, sparse and orderly. The hypertrophic index was reduced, since the fibroblastic density was lowered and collagen area density was decreased.

**Conclusion:**

HMME is an effective sonosensitizer and the combination of HMME-SDT treatment can exert significant benefits in reducing the formation of hypertrophic scars.

## Introduction

A hypertrophic scar, one type of pathological scars, is a persistent hyperplastic phenomenon at the scar site that occurs during the healing process of a dermal wound[1]. It is characterized by protrusion around normal skin, redness, hardness, itching and pain. Hypertrophic scars not only affect the appearance and functions of skin, but also can result in psychological burdens for the patient. They are unique to humans and occur in response to surgery, trauma, burns, or other forms of dermal injury and inflammatory conditions, and do not regress spontaneously[2]. Clinically, they are characterized by massive scar formation, which invades normal adjacent skin and extends beyond the region of the initial wound. Histologically, hypertrophic scars are characterized by exuberant dermal collagen formation with random orientation and assembly of the individual collagen fibers[3]. Uncontrolled fibroblast activity and excess collagen lead to overabundant extracellular matrix formation, and comprise the hallmark attributes of these tumors. Although they can be associated with pruritus and pain, the major complaint is the profound disfigurement that accompanies the uncontrolled growth of these lesions, particularly on the face. As a result, they can have a significant negative impact on the patients' quality of life.

Surgery combined with steroid injections, pressure dressings, silicone sheeting, cryotherapy, and laser excision represent the most conventional approaches used to treat hypertrophic scars, but fail to produce reliable and consistent results. Despite decades of work on developing approaches for treatment, there remain no consistent, reliable, and safe options available for this benign neoplasm. Classic surgical excision is notorious for its high recurrence rate, and with each recurrence, the size and extent of these lesions grow logarithmically [4]. Radiation therapy following surgical excision appears to offer the most reliable approach for recurring cases; however, the potential long-term consequences of ionizing radiation preclude its routine use, especially in younger patients. The best treatment is prevention in patients with a known pre-disposition [5]. It has come to our attention that sonodynamic treatment (SDT), when used in combination with surgery, rather than as a primary mode of treatment, may be used as an adjunctive therapy to treat hypertrophic scars. Accordingly, we hypothesize that SDT may be used to treat the surgical margins and surrounding region of tissue after surgical excision to minimize recurrence after hypertrophic scar excision, like that achieved using steroid injections, silicone sheeting, and pressure dressing. The application of SDT may reduce the formation of collagen and other extracellular matrix components. The prevention and treatment of hypertrophic scars represents a challenging problem in the field of modern medicine [6]. Therefore, it is of great significance to explore novel approaches for the treatment and prevention of hypertrophic scars.

In the present study, SDT, also named sonodynamic chemistry therapy (SDCT) [2] was used to treat hypertrophic scars on the surface of rabbit ears. On the basis of our previous studies, we combined the application of hematoporphyrin monomethyl ether (HMME) with SDT to explore the potential for synergistic treatment efficacy upon hypertrophic scars in rabbit ears.

## Materials and Methods

### Animals

All experimental procedures were in accordance to the Institutional Animal Care and Use Committee of Harbin Medical University, P.R. China. The protocol was approved by the Experimental Animal Ethic Committee of Harbin Medical University, China (Animal Experimental Ethical Inspection Protocol No. 2010102). The surgery procedures were performed under sodium pentobarbital anesthesia.60 healthy clean white rabbits were were used in this study.All animals were artificial fed according to the Specific Pathogen Free Animal Criteria, and were given food and water freely. Rabbits were purchased from the Veterinarian Institute of Heilongjiang Province and Shanghai Experimental Animal Center. The 60 rabbits(1.8–2.4 kg) were randomly divided into five equal groups: 1) untreated controls, 2) lesioned, 3) lesioned + HMME, 4) lesioned + US (Ultrasound), and 5) lesioned +HMME-SDT.

### Preparation of rabbit ear hypertrophic scar model

A modified rabbit ear hypertrophic scar model was established in the present study, according to procedures described previously by Morris[7]. After rabbits were housed individually for a period of one week, they were anesthetized by administration of sodium pentobarbital at a dose of 30 g/L via the ear vein. Under aseptic operation conditions, 4 round wounds each with a diameter of 6 mm were drilled on the ventral surface along the long axis of each ear. After this operation, the rabbits were provided with conventional anti-inflammatory treatment, and free access to food and water. At 21 days post-operation, the healed wound surface showed an obvious protrusion, which was an indicator of hypertrophic scar formation. At 42 days post-operation, the formation of hypertrophic scars reached their peak level.

### HMME-SDT

A multi-function physical therapy ultrasound device (Tianshi Technologies Ltd Co., Beijing, China) was used to generate ultrasounds at 1 MHz. Ultrasonic intensities (W/cm 2) were expressed as ISATA (spatial average/temporal average) measured by a stainless-steel ball radiometer (diameter 0.32 cm) over 9 s. In this study, the hypertrophic scars were immersed in degassed water, and placed directly in line with the center of ultrasonic transducer(diameter:2.5 cm; centre frequency:1.0 MHz; duty factor:10%; repetition frequency:100 Hz) surface at a distance of 3.0 cm, to ensure field uniformity. The internal surface of the glass tank was padded with ultrasound-absorbing materials to minimize reflection waves, the ultrasound transducer was placed in a 37°C water bath filled with degassed water and stained with black ink. HMME was provided by Yingfa Kangmei Ltd Co., (Beijing, China). Upon the full epithelialization of the wound on the surface of rabbit ears, the hypertrophic scars were subjected to HMME-SDT (1 W/cm2×90 s) through initial vein injection of HMME(20 µg/ml) followed by sonication 3 h later and once every two days for the experiment. Hypertrophic scars were assessed at 14, 28, 42 and 56 days post-lesion +/− treatment. The hypertrophic scars on the rabbit ears were placed in distilled water with a distance of approximately 3.0 cm away from the ultrasonic probe.

### General observation

General observations including growth status, color, texture and thickness of hypertrophic scars were recorded by taking pictures at designated time points. In addition, the height of hypertrophic scars was measured.

### Hypertrophic index (HI)

HE stained slices were examined under low magnification microscopy using a fiber measurement ruler and a hypertrophic index of the scars was calculated using the following expression formula: HI  =  A/B, where A  =  vertical height between the highest protrusion point of the scar and the surface of ear cartilage and B  =  vertical height between the surface of normal skin around the scar and the surface of ear cartilage.

### Specimen detection

During epithelialization on days 14, 28, 42 and 56 post-operation, 8 samples were harvested from five groups: 1) untreated controls, 2) lesioned, 3) lesioned + HMME, 4) lesioned + US (Ultrasound), and 5) lesioned +HMME-SDT. Totally, 160 specimens were harvested and fixed with 4% paraformaldehyde for 48 h. Routine dehydration, paraffin embedding and serial sectioning with a thicknesses of 3–5 µm were sequentially performed on these specimens. Specimens subjected to HE and VG staining were examined under the microscope.

### Fibroblast density

In these HE stained slices, a total of 10 vision areas from each part of the scar, such as central shallow layer, central deep layer and both side locations of the scar, were randomly selected for microscopic examination at 400×. The number of fibroblasts was recorded to calculate a fibroblast density in unit area.

### Collagen tissue density

In VG stained slices, a total of 10 vision areas from each part of the scar, such as central shallow layer, central deep layer and both side locations of the scar slices, were randomly selected for microscopic examination at 400×. The density of collagen was recorded to calculate a collagen density in unit area.

### Statistical Analysis

The SPSS Version 13.0 for Windows (SPSS Inc., Chiacago, Illinois) was used for data analysis. Data are presented as mean ± SD. The Student's t-test was used to test for differences between the lesioned and lesioned +HMME-SDT groups. ANOVA is performed for overall analyses among these three groups.The X^2^ test and rank -order test were used for statistical analysis. A P-value<0.05 was considered to be statistically significant.

## Results

### General observation

**Figure 1:**
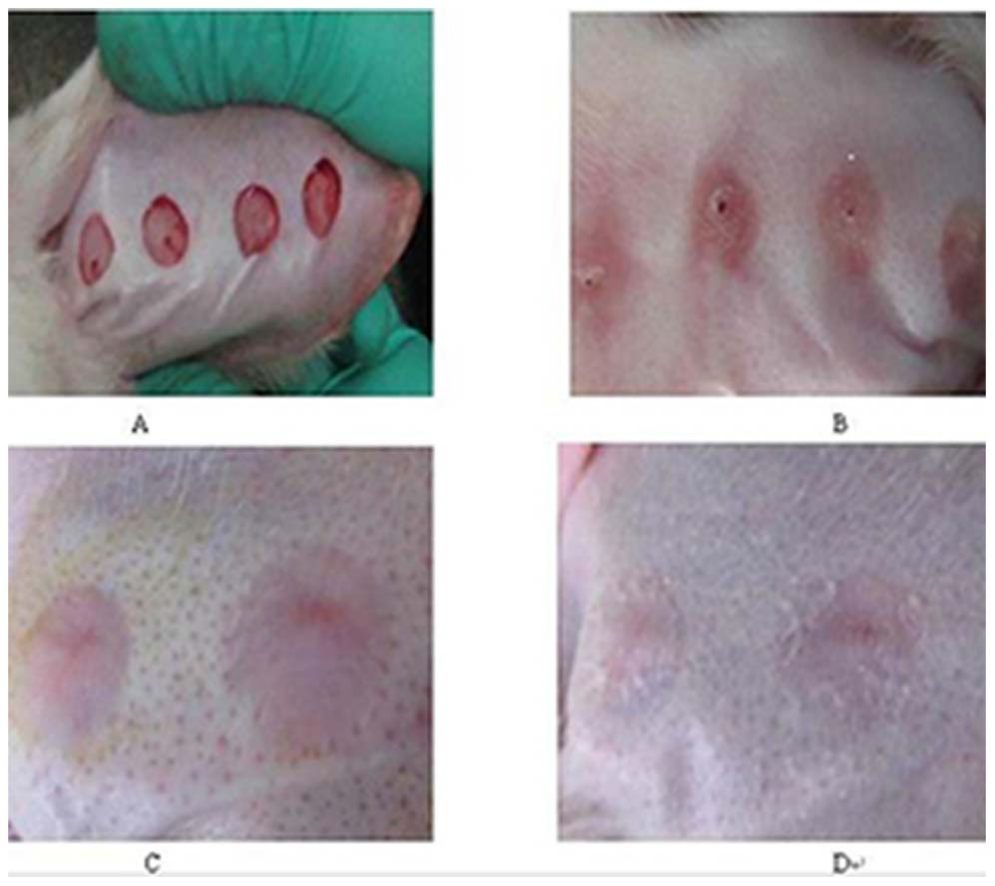
The genernal observation after HMME-SDT on hypertrophic scarring. United States of America A: Hypertrophic scar model; B: Scars following epithelialization for 42 days ; C: Scars following epithelialization for 56 days; D: Scars following epithelialization for 56 days in the lesioned +treatment group.

Following wound introduction, scars were gradually generated from the wound area and with the falling of scab skin, the development of hypertrophic scars was observed. In our model, wound healing was observed after 12–14 days, and hard scleroma could be touched after 20–24 days. With the development of hypertrophic scars, the scleroma with pale red color was obviously raised above the surface of normal skin. Although the hyperplasia did not exceed the scope of the original wound, the scleroma was 2-fold the thickness of the rabbit ear skin. Hypertrophic scars reached their peak level at 42 days after epithelialization. At this stage, the scar thickness was approximately 3-fold the skin thickness of the rabbit ear skin. In the lesioned +HMME-SDT group, during drug administrations, the scars revealed a gradual softening trend and diminished volume. With the extension of treatment duration, the scars exhibited a flat shape and faded color. (Fig. 1).

### Histological observation

**Figure 2:**
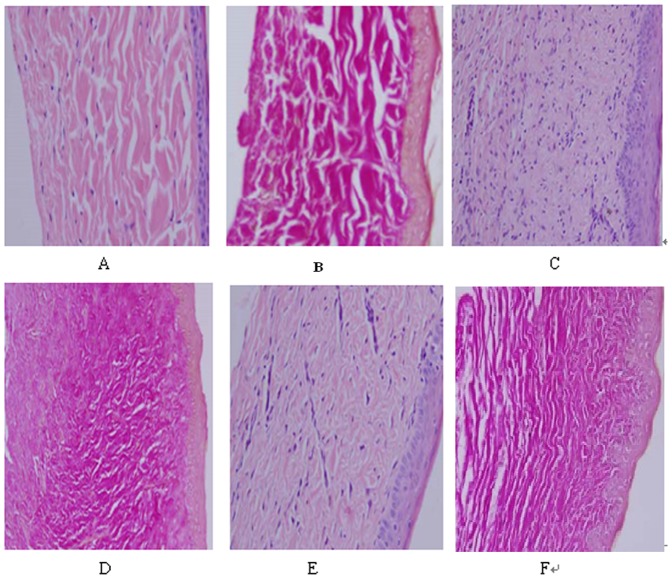
The histological observation after HMME-SDT on hypertrophic scarring. A: Untreated control group HE staining×200; B: Untreated control group VG staining×200; C:Lesioned group 56 days after epithelialization HE staining×200; D:Lesioned group 56 days after epithelialization, VG staining×200 ; E:Lesioned + Treatment group 56 days after epithelialization, HE staining×200;F:Lesioned + Treatment group 56 days after epithelialization, VG staining×200

In the lesioned group, dermal tissue revealed obvious hyperplasia, thickening, and a large number of fibroblasts, collagen tissue and blood vessels and disarranged collagen with nodule or vortex-like distribution. With the development of hypertrophic scars, no significant changes in these parameters were observed. In contrast, scar tissue, in the lesioned +HMME-SDT group, revealed a gradual reduction in blood vessels, fibroblasts and collagen fibers, as well as pale red, sparse, and ordered arrangement of their collagen fibers. (Figure 2)

### HI comparison

Compared with the lesioned group, a reduced HI was observed in the lesioned +HMME-SDT group. At 28 days after epithelialization, a significant difference in HI was obtained between the lesioned and lesioned +HMME-SDT groups (P<0.01). These differences in HI scores between the lesioned and lesioned +HMME-SDT groups increased further with the extension of treatment time as shown in Table 1. It is worth mentioning that at 56 days after epithelialization, a minor difference in HI was obtained between the lesioned and lesioned +US groups (P<0.064).

**Table 1:** Effect of HMME-SDT on HI of rabbit ear hypertrophic scars.

group	n	14 days after epithelialization	28 days after epithelialization	42 days after epithelialization	56 days after epithelialization
untreated controls	12	1.01±0.13	1.13±0.21	0.99±0.54	1.21±0.21
lesioned +HMME	12	2.68±0.19Δ	3.40±0.42Δ	3.56±0.24Δ	3.26±0.45Δ
lesioned+US	12	2.75±0.35ΔΔ	3.05±0.24ΔΔ	3.18±0.23ΔΔ	2.98±0.19ΔΔ
lesioned	12	2.71±0.23*	3.31±0.59*	3.65±0.49*	3.31±0.55*
lesioned+HMME-SDT	12	2.51±0.29	2.21±0.28**	2.18±0.38**	1.45±0.18**

Note: *Compared with the untreated controls P<0.01; **Compared with the lesioned P<0.01; ΔCompared with the lesioned P>0.05 ; ΔΔCompared with the lesioned P = 0.064; ΔΔCompared with the lesioned+HMME P>0.05.

### Fibroblast density

Compared with the untreated control group, the fibroblast density in the lesioned group was significantly higher and revealed a gradually decreasing trend after achieving a peak level at 28 days after epithelialization. In contrast, HMME-SDT treatment resulted in a significant reduction of fibroblast density as compared with the lesioned group. (Table 2)

**Table 2:** Effect of HMME-SDT on fibroblast density of rabbit ear hypertrophic scars.

group	n	14 days after epithelialization	28 days after epithelialization	42 days after epithelialization	56 days after epithelialization
untreated controls	12	26.12±4.65	24.12±3.43	22.32±4.13	24.99±4.12
lesioned +HMME	12	72.68±0.21Δ	73.58±0.39Δ	73.75±0.42Δ	73.47±0.25Δ
lesioned+US	12	77.48±3.56ΔΔ	76.25±5.05ΔΔ	75.34±4.01ΔΔ	61.58±3.24ΔΔ
lesioned	12	75.99±4.87*	89.54±8.64*	76.43±6.47*	62.12±4.08*
lesioned+HMME-SDT	12	64.54±4.14	71.34±5.12**	54.32±5.21**	35.43±3.98**

Note: * Compared with the untreated controls P<0.01;**Compared with the lesioned P<0.05 ; ΔCompared with the lesioned P>0.05 ; ΔΔ Compared with the lesioned+HMME P>0.05.

### Collagen fiber area density

The area density of collagen fibers in the lesioned group was significantly higher than that in the untreated control group. At 28 days after epithelialization peak levels, in the area density of collagen fibers were present followed by a gradual reduction thereafter. HMME-SDT treatment significantly reduced the area density of collagen fibers at 28 days after epithelialization and these differences between the lesioned and lesioned +HMME-SDT groups remained present for the duration of the experiment. (Table 3)

**Table 3:** Effect of HMME-SDT on area density of collagen fibers in rabbit ear hypertrophic scars.

group	n	14 days after epithelialization	28 days after epithelialization	42 days after epithelialization	56 days after epithelialization
untreated controls	12	25.78±4.42	23.67±3.87	23.98±4.87	24.85±4.61
lesioned +HMME	12	72.48±0.24Δ	86.45±0.75Δ	73.74±0.78Δ	63.78±0.47Δ
lesioned+US	12	73.26±3.48ΔΔ	85.75±5.47ΔΔ	71.25±4.89ΔΔ	65.47±3.78ΔΔ
lesioned	12	74.98±4.86*	89.87±7.21*	74.45±5.24*	66.58±4.24*
lesioned+HMME-SDT	12	66.23±4.13	71.98±4.02**	47.78±5.09**	33.13±3.09**

Note: *Compared with the untreated controls P<0.01;**Compared with the lesioned P<0.05; ΔCompared with the lesioned P>0.05 ; ΔΔ Compared with the lesioned+HMME P>0.05.

## Discussion

Hypertrophic scars represent a type of hypertrophic disease of fiber tissue that occurs during the healing process of a wound in skin tissue. Current therapeutic strategies for hypertrophic scars are not ideal, therefore, clinical research in this area has become a hot topic [4]. It has been reported that SDT exerts a significant role in the induction of apoptosis and inhibition of cell proliferation[8]. However, its mechanisms for the prevention of hypertrophic scars and feasibility for the treatment of hypertrophic scars remains unclear[2]. In our previous study, we demonstrated that SDT plays an important role in the pathogenesis of excessive proliferation of fibroblasts[12]. Recently, SDT has been identified as a novel treatment for cancer. In this regard, SDT has the capacity to focus its energy upon specific parts of the tumor [9] and, over time, the activation of sonosensitizer enrichment within the tumor produces little damage to normal tissue around the tumor; as a result, SDT has excellent application prospects in the treatment of cancer [10]. However, use of SDT in the treatment of hypertrophic scars has rarely been reported. In the present study, we explored the treatment efficacy of SDT on rabbit ear hypertrophic scars in the presence of HMME as a sonosensitizer.

In this study we first established a model for induction of hypertrophic scars. In our model, an obvious hyperplasia on rabbit ears was observed and characteristics of protrusion above skin surface with a 2-fold increase in the thickness of normal skin and the color of pale red was observed within 21 days after lesion. This scar formation reached a peak level at 42 days after epithelialization at which time the hypertrophic scars revealed a 3-fold increase in the thickness of normal skin. HMME-SDT treatment resulted in the gradual softening and volume diminution of the scar, eventually becoming flat in shape and faded color with continued treatment. In lesioned specimens, HE and VG staining revealed obvious hyperplasia and thickening of dermal tissue, the accumulation of a large number of fibroblasts, collagen tissue and blood vessels. The disordered arrangement of collagen fibers showed nodule or vortex-like distribution, and no obvious change over time during the formation of hypertrophic scars. In contrast, the group receiving HMME-SDT treatment showed a gradual reduction in blood vessels, fibroblasts and collagen fibers with pale red color and a sparse and ordered arrangement in scar tissue were observed over the treatment time period. In the HMME-SDT group, the HI was obviously lower when compared with the untreated lesion group. At 28 days after epithelialization, HI scores between the lesioned and lesioned +HMME-SDT groups revealed a significant difference. It is worthy of mention that mentioning that at 56 days after epithelialization, a minor difference in HI was obtained between the lesioned and lesioned +US groups. This shows that ultrasound alone has minor effects, but is not statistically significant. The fibroblast density in the lesioned group was significantly higher than that in the untreated control group, while HMME-SDT treatment significantly reduced the density of fibroblasts as compared with that obtained in the lesioned group. The area density of collagen fibers in the lesioned group was significantly higher than that in the normal group, and reached a peak level at 42 days after epithelialization. With HMME-SDT treatment the area density of collagen fibers was reduced significantly at 28 days after epithelialization. On the basis of the data obtained from HI, fibroblast density and area density of hypertrophic scars on rabbit ears as well as HE and VG staining results, it is clear that HMME-SDT treatment can serve as an effective means to alleviate the degree of scar proliferation on rabbit ears. Such beneficial effects are due to the capacity for HMME-SDT treatment to reduce the number of fibroblasts and content of collagen fibers in hypertrophic scars, thus realizing the sparse and ordered arrangement of collagen fibers[11]. While additional work will be required to fully understand the mechanisms involved with HMME-SDT treatment, the present results demonstrate the ability for this treatment to effectively reduce the proliferation of fibroblasts and synthesis of collagen in hypetrophis scars, thus offering a promise for clinical application.
